# Insulin-like growth factor binding protein 5b of *Trachinotus ovatus* and its heparin-binding motif play a critical role in host antibacterial immune responses *via* NF-κB pathway

**DOI:** 10.3389/fimmu.2023.1126843

**Published:** 2023-02-14

**Authors:** Hehe Du, Yongcan Zhou, Xiangyu Du, Panpan Zhang, Zhenjie Cao, Yun Sun

**Affiliations:** ^1^ State Key Laboratory of Marine Resource Utilization in South China Sea, Hainan University, Haikou, China; ^2^ Hainan Provincial Key Laboratory for Tropical Hydrobiology and Biotechnology, College of Marine Science, Hainan University, Haikou, China; ^3^ Collaborative Innovation Center of Marine Science and Technology, Hainan University, Haikou, China

**Keywords:** IGFBP5, HBM, subcellular localizations, antibacterial immunity, NF-κB pathway

## Abstract

**Introduction:**

Insulin-like growth factor binding protein 5 (IGFBP5) exerts an essential biological role in many processes, including apoptosis, cellular differentiation, growth, and immune responses. However, compared to mammalians, our knowledge of IGFBP5 in teleosts remains limited.

**Methods:**

In this study, TroIGFBP5b, an IGFBP5 homologue from golden pompano (*Trachinotus ovatus*) was identified. Quantitative real-time PCR (qRT-PCR) was used to check its mRNA expression level in healthy condition and after stimulation. *In vivo* overexpression and RNAi knockdown method were performed to evaluate the antibacterial profile. We constructed a mutant in which HBM was deleted to better understand the mechanism of its role in antibacterial immunity. Subcellular localization and nuclear translocation were verified by immunoblotting. Further, proliferation of head kidney lymphocytes (HKLs) and phagocytic activity of head kidney macrophages (HKMs) were detected through CCK-8 assay and flow cytometry. Immunofluorescence microscopy assay (IFA) and dual luciferase reporter (DLR) assay were used to evaluate the activity in nuclear factor-κB (NF-κβ) pathway.

**Results:**

The TroIGFBP5b mRNA expression level was upregulated after bacterial stimulation. *In vivo*, TroIGFBP5b overexpression significantly improved the antibacterial immunity of fish. In contrast, TroIGFBP5b knockdown significantly decreased this ability. Subcellular localization results showed that TroIGFBP5b and TroIGFBP5b-δHBM were both present in the cytoplasm of GPS cells. After stimulation, TroIGFBP5b-δHBM lost the ability to transfer from the cytoplasm to the nucleus. In addition, rTroIGFBP5b promoted the proliferation of HKLs and phagocytosis of HKMs, whereas rTroIGFBP5b-δHBM, suppressed these facilitation effects. Moreover, the *in vivo* antibacterial ability of TroIGFBP5b was suppressed and the effects of promoting expression of proinflammatory cytokines in immune tissues were nearly lost after HBM deletion. Furthermore, TroIGFBP5b induced NF-κβ promoter activity and promoted nuclear translocation of p65, while these effects were inhibited when the HBM was deleted.

**Discussion:**

Taken together, our results suggest that TroIGFBP5b plays an important role in golden pompano antibacterial immunity and activation of the NF-κβ signalling pathway, providing the first evidence that the HBM of TroIGFBP5b plays a critical role in these processes in teleosts.

## Introduction

The insulin-like growth factor (IGF) system is mainly composed of IGF-I/II, IGF receptors, and IGF-binding proteins (IGFBPs) (1). IGFBPs act as IGF carriers and regulate their biological distribution (2). The IGF signaling pathway has been proven to be crucial for the onset and progression of numerous diseases as well as the control of cellular activities (1, 3). Given the extensive evidence regarding the significance of IGF, the IGFBP family, which was identified and designated IGFBP1 to IGFBP6, has attracted much attention in recent years (4, 5). IGFBP5 belongs to one of the most diverse groups in the biologically active IGFBP family (6). It was first found in human bone extracts (7). Since then, IGFBP5 has been cloned from a wide range of species and has the highest level of sequence similarity among the IGFBP family (8–10).

In mammals, IGFBP5 plays a variety of functions in cellular activities and has been reported to be associated with many diseases (1, 4). Studies found that the expression of IGFBP5 may stimulate retinal pigment epithelium (RPE) cell fibrosis, leading to the progression of proliferative vitreoretinopathy (11, 12). It was discovered that the expression of IGFBP5 was downregulated in kidney renal papillary renal cell carcinoma patients, could strengthen tissue regeneration, and had an anti-inflammatory effect by maintaining immune homeostasis (13–15).

Overall, IGFBP5 remains poorly understood in teleosts compared with mammals. To date, few studies have focused on the immune response of the IGF system in fish, and even fewer ones have focused on the function of IGFBP5. Several IGFBP5 sequences in fine flounder (*Paralichthys adspersus*), zebrafish (*Danio rerio*), grass carp (*Ctenopharyngodon idella*), salmon, and rainbow trout (*Oncorhynchus mykiss*) have been cloned and characterized (8, 16–19). These studies mainly focused on the aspects of evolution, function in growth, muscle or embryonic development, and hormonal regulation (20, 21).

IGFBP5 contains a nuclear localization signal (NLS) in the C-terminal domain which is suggested to help IGFBP5 translocate to the nucleus and activate many transcription factors in the nucleus of cells involved in immune and inflammatory reactions (22, 23). Recently, many studies have focused on IGFBP5 nuclear trafficking and demonstrated that its subcellular compartmentalization affects its functions—for example, NLS-mutated IGFBP5 is mainly located in the cytoplasm, and it can enhance proliferation and migration (24). IGFBP5 induces Egr-1 and binds to each other in the nucleus, resulting in the promotion of fibrotic gene transcription (25). Moreover, according to some reports, the NLS domain contains a heparin-binding motif (HBM; ^206^KRKQCK^211^) that appears to be key in determining the various functions of IGFBP5 (26–29).

*Vibrio harveyi* is the main threat to the large-scale farming of *Trachinotus ovatus*, an important commercial fish in China (30, 31). In the present study, TroIGFBP5b was cloned and identified, and its different expression patterns were examined. To assess whether HBM deficiency affects its subcellular location and influences its function, we generated a mutant containing a truncated form of TroIGFBP5b in which the HBM motif of the NLS domain was deleted. The findings provide insight into the mechanisms underlying the immune function of TroIGFBP5b.

## Materials and methods

### Fish and cells

*T. ovatus* (average weight 18.5 g) from Hainan Province was temporarily reared for 1 week before the experiments. Golden pompano snout (GPS) cells, kindly provided by Professor Qin, were cultured in L-15 medium [containing 10% fetal bovine serum (FBS, Gibco), 100 U/ml penicillin, and 100 U/ml streptomycin] at 26°C (32). Human embryonic kidney (293T) cells were incubated in Dulbecco’s modified Eagle’s medium with 10% FBS and incubated at 37°C (5% CO_2_ incubator).

### Pathogenic bacteria and challenge experiment

*V. harveyi* that was isolated by our laboratory from golden pompano was used as the pathogen and cultured in Luria–Bertani (LB) medium (containing 100 μg/ml ampicillin, Amp) at 30°C (31). The suspension was diluted to 3 × 10^7^ colony-forming units (CFU)/ml when the OD_600_ value reached approximately 0.6. The fish were intraperitoneally injected with 0.1 ml of the suspension, and the same volume of phosphate-buffered saline (PBS) was injected as the control. The liver, spleen, and head kidney were collected at 6, 9, 12, and 24 h post-infection (hpi). Three separate samples were prepared as well.

### Quantitative real-time PCR

Total RNA of tissues and cells was extracted using E.A.N.A. Total RNA Extraction Kit (OMEGA, USA) and digested with DNase (OMEGA, USA). cDNAs were synthesized using Eastep^®^ RT Master Mix Kit (Promega, USA). qRT-PCR was performed to quantify the target gene mRNA level using SYBR ExScript qRT-PCR Kit (Promega, China). *Beta-2-microglobulin* was used as the housekeeping gene, and data were analyzed by the 2^-ΔΔCT^ method (33). The primers used are listed in **Supplementary Table S1**.

### Gene cloning and analysis

The full open reading frame of the TroIGFBP5b sequence was amplified with primers TroIGFBP5b-F1/TroIGFBP5b-R1 by using *T. ovatus* liver cDNA as the template. The TroIGFBP5b sequence was blasted at the National Center for Biotechnology Information (NCBI, http://www.ncbi.nlm.nih.gov/blast). The structural domain was predicted at SMART online (http://smart.embl-heidelberg.de/). The three-dimensional (3D) structure prediction of TroIGFBP5b was carried out on the SWISS-MODEL website, and the visualization of the predicted protein 3D structure was achieved using PyMOL software. The phylogenetic tree constructed by MEGA 7.0 employed the neighbor-joining (NJ) method.

### Plasmid construction and small interfering RNA synthesis

The TroIGFBP5b sequence, except for the signal peptide (SP) domain, was amplified with primer TroIGFBP5b-F2/TroIGFBP5b-R1, using the pEASY-T-TroIGFBP5b plasmid as a template by PCR, and named TroIGFBP5b-ΔSP. Subsequently, two HBM-deleted mutants (^215^RKGFFKRKQCKPSRGRKR^232^ to ^215^RKGFFPSRGRKR^226^, delete ^220^KRKQCK^225^) of TroIGFBP5b were amplified with full-length TroIGFBP5b or TroIGFBP5b-ΔSP using the primer pairs TroIGFBP5b-F1/TroIGFBP5b-R3 or TroIGFBP5b-F2/TroIGFBP5b-R3 and TroIGFBP5b-F3/TroIGFBP5b-R1 by overlap PCR assay and named TroIGFBP5b-ΔHBM and TroIGFBP5b-Δ(HBM+SP), respectively.

To construct a eukaryotic expression vector for overexpressing IGFBP5 *in vivo*, TroIGFBP5b and TroIGFBP5b-ΔHBM were inserted into the pCN3 vector, which expresses the human cytomegalovirus immediate-early promoter, at the *EcoR* V site, resulting in pTroIGFBP5b and pTroIGFBP5b-ΔHBM (34). pEGFPX-N3 was used for subcellular localization and was reformed from the pEGFP-N3 vector (35). Recombinant GFP plasmids were constructed by connecting TroIGFBP5b, TroIGFBP5b-ΔSP, TroIGFBP5b-ΔHBM, and TroIGFBP5b-Δ(HBM+SP) to pEGFPX-N3 at the *Sma*I site, resulting in pTroIGFBP5b-WT-N3, pTroIGFBP5b-ΔHBM-N3, pTroIGFBP5b-ΔSP-N3, and pTroIGFBP5b-Δ(HBM+SP)-N3, respectively. To obtain biologically active recombinant TroIGFBP5b-ΔSP and TroIGFBP5b-Δ(HBM+SP) proteins, pET-32a, which could express a His-tag and a thioredoxin protein (Trx), was used and linearized at the *Eco*RV site. All of the above-mentioned positive constructs were confirmed by colony PCR and sequencing. The plasmids used in the cell-related experiments, as well as those injected into the fish body, were endotoxin-free plasmids harvested using Plasmid Extraction Kit (TransGen, China) according to the supplier’s instructions.

The siRNA synthesis followed the instructions of RiboMAX™ Express RNAi System (Promega, USA) as described (36). Briefly, two pairs of primers, siTroIGFBP5b-P1/siTroIGFBP5b-P2 and siTroIGFBP5b-P3/siTroIGFBP5b-P4, were designed to obtain two DNA oligonucleotides after incubation at 95°C for 5 min. The templates were allowed to cool slowly to room temperature (RT). Next, these two DNA oligonucleotides were used to separately synthesize the sense strand RNA or the antisense strand RNA templates at 37°C for 2 h. Afterwards, the DNA template was removed from the separate short RNA strands by digestion with DNase, and then the two RNA strands were mixed to synthesize the siRNA. Finally, the synthesized siRNA was purified following the manufacturer’s instructions. The control siRNA (siTroIGFBP5b-C) was synthesized with siTroIGFBP5b- C-P1/P2 and siTroCCL4-C-P3/P4 as described above. The primers used in this study are listed in **Supplementary Table S1**.

### *In vivo* overexpression and knockdown of TroIGFBP5b

To evaluate the *in vivo* role of TroIGFBP5b, the fish were intramuscularly injected with 15 μg (0.1 ml) overexpression plasmids (pTroIGFBP5b and pCN3). The knockdown of TroIGFBP5b was achieved by an intramuscular injection of 15 μg siTroIGFBP5b or siTroIGFBP5b-C into the fish. For these experiments, 0.1 ml PBS was injected as a control. To further study the function of HBM on bacterial infection *in vivo*, the pTroIGFBP5b-ΔHBM plasmid was also injected into the fish as described above, and the pTroIGFBP5b, pCN3, and PBS groups were repeated to compare differences.

### *In vivo* antibacterial ability assay

After the post-injection of overexpressing plasmids for 5 days and the post-injection of siRNA for 12 h, 0.1 ml of *V. harveyi* (2 × 10^7^ CFU/ml) suspension was injected into all groups intraperitoneally. The appropriate size of the liver, spleen, and head kidney tissue blocks was determined aseptically at 6, 9, and 12 hpi. The weighed tissue blocks were ground in 300 µl PBS, and 700 µl PBS was added. In total, 100 µl homogenate was spread evenly onto LB plates (containing 100 μg/ml Amp) to measure the bacterial loads of the different tissues. Finally, the number of colonies per gram was calculated.

### Subcellular localization

GPS cells were cultivated to analyze the subcellular localization of TroIGFBP5b according to the method of Chen et al. (33). Briefly, before transfection, GPS cells were grown in six-well plates overnight until reaching 60% confluence. pTroIGFBP5b-WT-N3, pTroIGFBP5b-ΔHBM-N3, pTroIGFBP5b-ΔSP-N3, pTroIGFBP5b-Δ(HBM+SP)-N3, and pEGFPX-N3 (4 μg) were transfected into GPS cells using LipoFiter3.0 (Hanbio, Shanghai, China). At 48 h post-transfection, the cells were fixed for 15 min with 4% (v/v) paraformaldehyde at RT. 4′,6-Diamidino-2-phenylindole (1 μg/ml) was used to stain the nuclei for 20 min, and rhodamine B was used to highlight the whole cell. Finally, the cells were washed with PBS until the cleaning solution became colorless, and the cells were observed using an inverted microscope. To monitor the localization of TroIGFBP5b after stimulation, *V. harveyi* (1 × 10^5^ CFU/ml) or lipopolysaccharide (LPS; 100 ng/ml) was added to the transfected cells (TroIGFBP5b-WT and TroIGFBP5b-ΔHBM) for 4 h after transfection for 48 h. The ensuing steps were the same as those described above.

### Protein expression and purification

Recombinant TroIGFBP5b (rTroIGFBP5b), HBM mutant (rTroIGFBP5b-ΔHBM), and rTrx were purified as described in a previous report (33). After exploring the induction conditions, the optimum induction condition was determined by incubating at 20°C for 8 h after adding isopropyl β-d-1-thiogalactopyranoside (0.5 mM). After purification by a Ni Sepharose column, PBS was used to dialyze the recombinant proteins which were concentrated with PEG8000. The concentration of the purified recombinant protein was measured by the Bradford method.

### Phagocytic activity detection of head kidney macrophages through flow cytometry

*T. ovatus* head kidneys were collected and rinsed with PBS three times aseptically. Density gradient centrifugation was used to obtain head kidney macrophages (HKMs) from *T. ovatus* as previously described with Percoll (GE Healthcare, USA) (37). The isolated cells were added to a six-well plate (1 × 10^7^ cells/well) containing L-15 medium for 2 h, and then 100 μl each of 200 μg/ml rTroIGFBP5b, rTroIGFBP5b-ΔHBM, and rTrx was infused into each well; the mixture was incubated at 26°C overnight. Green fluorescent microspheres (Aladdin, China) were diluted to 1% (w/v) L-15 (containing 10% FBS) medium and then added to each well at 26°C in the dark for 2 h. Before being subjected to Guava easyCyte™ Flow Cytometer (Millipore, USA), the cells were washed and resuspended in L-15 to 1 × 10^9^ cells/ml. The experiment was performed in triplicate.

### Proliferation detection of head kidney lymphocytes by CCK-8 assay

*T. ovatus* head kidneys were collected and rinsed aseptically with PBS three times. Density gradient centrifugation was used to obtain head kidney lymphocytes (HKLs) from *T. ovatus* as previously described by Percoll (GE Healthcare, USA) (36). The prepared HKLs resuspended in L-15 medium with 10% FBS were added (90 μl) to a 96-well culture plate (1 × 10^5^ cells/well). Subsequently, 10 μl of rTroIGFBP5b and rTroIGFBP5b-ΔHBM (final concentrations of 10, 50, 100, 200, and 300 μg/ml) or rTrx (300 μg/ml) was incubated for 12 h at 26°C, and PBS was added as a control. As per the instructions, Cell Counting Kit-8 (CCK8) (Hanbio, Shanghai, China) was applied to measure the proliferation of HKLs. The results were calculated as follows: (*A*_450_ of protein-treated cells − *A*_450_ of the empty well)/(*A*_450_ of control cells − *A*_450_ of the empty well). The empty well contained only medium and CCK-8 solution. The experiment was repeated in triplicate.

### Immunofluorescence microscopy assay

293T cells were transfected with pTroIGFBP5b, pTroIGFBP5b-ΔHBM, and pCN3 as described above. The immunofluorescence microscopy assay was performed as previously described (38). The primary antibody used was an anti-p65 polyclonal antibody (Bioss, Beijing, China) at 1/200 dilution.

### Western blot assay

GPS cells were transfected with pTroIGFBP5b-WT-N3, pTroIGFBP5b-ΔHBM-N3, pTroIGFBP5b-ΔSP-N3, pTroIGFBP5b-Δ(HBM+SP)-N3, or pEGFPX-N3 in 10-cm-diameter culture dishes. Nuclear and Cytoplasmic Extraction Reagent Kit (Beyotime, Beijing, China) was used to separately extract nuclear and cytoplasmic proteins. After protein separation by 15% SDS-PAGE and transfer to a PVDF membrane (Millipore, Germany), the membrane was blocked with 5% BSA for 1 h. Then, the membrane was incubated with anti-EGFP (1/2,000 dilution, Bioss, Beijing, China) at 4°C overnight. After 10 min of washing with TBST three times, the secondary antibody (HRP-conjugated goat anti-mouse IgG and 1/2000 dilution) was added and incubated for 1 h at RT. Anti-β tubulin and anti-Histone H3 (Bioss, Beijing, China) were used as the nuclear and cytoplasmic internal references, respectively. The experiment was performed in triplicate.

### Dual-luciferase reporter assay

A DLR assay was performed to examine the activation of NF-κB. GPS and 293T cells (1 × 10^6^ cells/well) were seeded in a 24-well plate. A total of 0.2 mg of NF-κB-specific firefly luciferase reporter vector, the pGL4.32 vector (luc2P/NF-κB, Promega, USA), 0.05 mg of pRL-CMV (a control vector), and 0.2 mg of pTroIGFBP5b or pTroIGFBP5b-ΔHBM were co-transfected into cells using LipoFiter3.0 (Hanbio, Shanghai, China). After transfection for 48 h, the firefly and Renilla luciferase activities in the cell lysates were measured using Dual-Luciferase Reporter Assay Kit (Promega, USA). All experiments were conducted three times independently.

### Statistical analysis

All data in this study were statistically processed using GraphPad Prism (version 8.0.2). Statistically significant differences were evaluated by the *t*-test, with the *P*-value indicated (*P < 0.05, **P < 0.01).

## Results

### Sequence characterization of TroIGFBP5b

**Figure 1:**
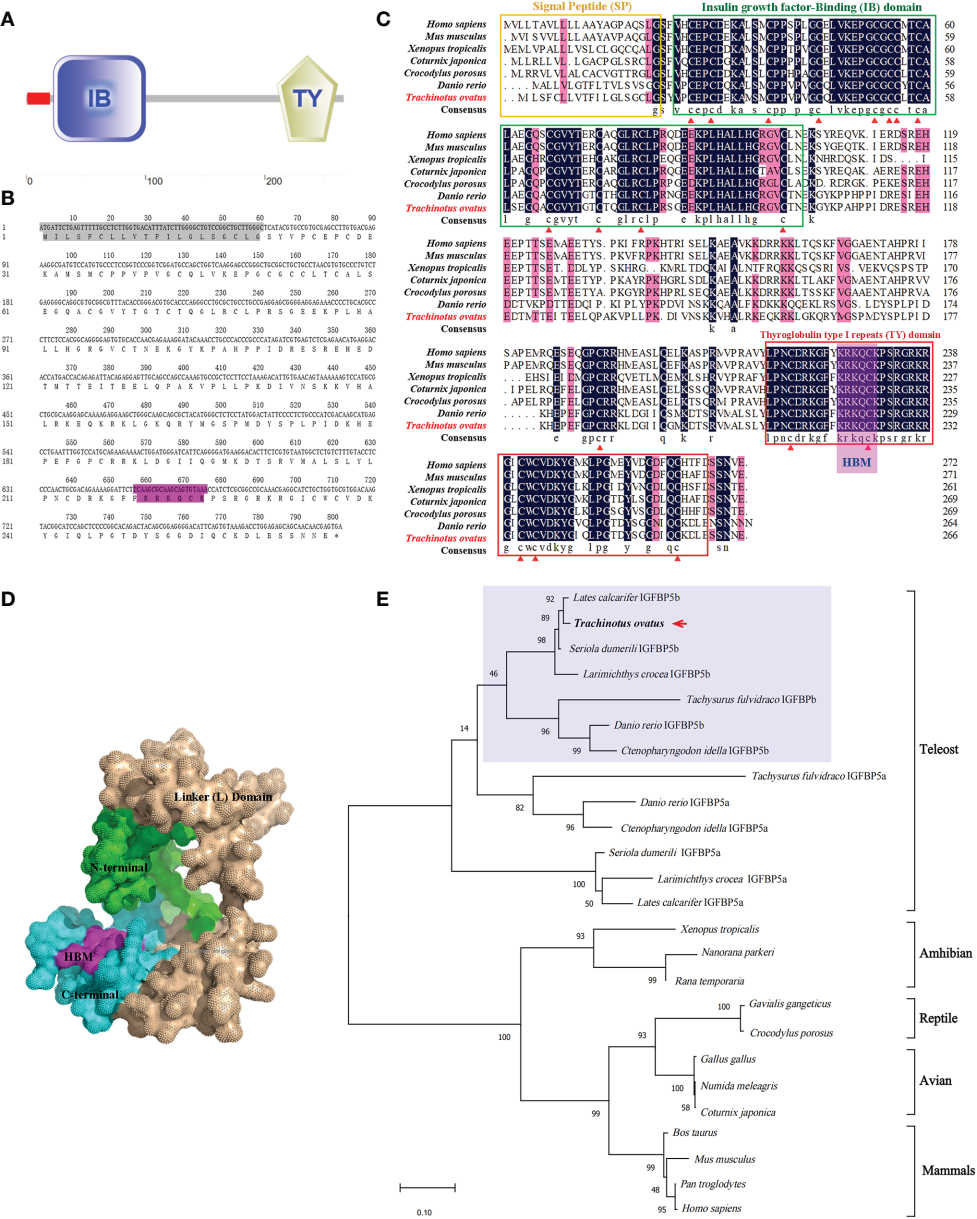
Predicted domains, multiple alignments, and phylogenetic tree for TroIGFBP5b amino acid sequence. **(A)** The domains were predicted by SMART. The red box was a signal peptide (SP) as detected by the SignalP v4.0 program. **(B)** The TroIGFBP5b sequence **in** the background show**n** in gr**a**y was the SP, and the purple color indicated the heparin-binding motif (HBM) sequence. **(C)** Multiple sequence alignment analyses of TroIGFBP5b. The consensus and ≥75% identical residues were in black and pink among the aligned sequences. The putative SP, insulin growth factor-binding, and thyroglobulin type I repeat regions were marked by the yellow, green, and red boxes, respectively. The red triangles indicated the 18 conserved cysteine residues. **(D)** Surface view of the TroIGFBP5b 3D structure. The N-terminal and C-terminal were marked with green and blue colors, respectively, and the purple region represented the HBM. **(E)** Phylogenetic tree. The selected protein sequences are listed in **Table 1**. The newly characterized TroIGFBP5b was marked with an arrow.

*TroIGFBP5b* is 801 bp in length and encodes 266 amino acids (a.a.) (NCBI GenBank accession number OP712620). According to SMART prediction analysis, TroIGFBP5b is composed of a signal peptide (SP) (1–20 a.a.), an insulin growth factor-binding (IB) domain (23–100 a.a.), and a thyroglobulin type I repeat (TY) domain (210–261 a.a.) (**Figure 1A**). In addition, TroIGFBP5b contains a highly conserved HBM (220–226 a.a.) in the NLS sequence (215–232 a.a.) of the C-terminal domain (**Figure 1B**). The sequence alignment analysis showed that the IB and TY domains of TroIGFBP5b were very highly conserved among vertebrates, including mammals (*Homo sapiens* and *Mus musculus*), amphibians (*Xenopus tropicalis*), reptiles (*Chelonia mydas*), avians (*Gallus gallus)*, and teleosts (*D. rerio*), suggesting its conservation during species evolution. The N-domain contains 12 conserved cysteine residues, and the C-domain contains six, which contributed to the structural stability by intradomain disulfide bonds between cysteine residues (**Figure 1C**). From the 3D predicted structure, HBM is located in the cavity formed by the C- and N- termini (**Figure 1D**). Identities with mammals, avians, reptiles, and amphibians are relatively lower, ranging from 51.66% to 58.74%. According to the multiple sequence alignment analysis, identities vary in teleosts, ranging from 60.15% to 97.38%. TroIGFBP5b shows a high identity with IGFBP5b in *Seriola dumerili* (97.38%), *Lates calcarifer* (94.91%), and *Larimichthys crocea* (92.54%) (**Table 1**). A phylogenetic tree was constructed using the NJ algorithm, showing that TroIGFBP5b clusters with IGFBP5b of other teleosts and resembles *Lates calcarifer* IGFBP5b, the closest phylogenetically, with a bootstrap value of 92 (**Figure 1E**).

**Table 1:** Identities of TroIGFBP5b with other species.

Species	Accession number	Identities (%)
*Crocodylus porosus*	XP_019409774.1	51.66
*Gavialis gangeticus*	XP_019378195.1	51.85
*Pan troglodytes*	XP_003309513.1	53.85
*Homo sapiens*	NP_000590.1	53.85
*Xenopus tropicalis*	XP_031748507.1	54.28
*Bos taurus*	NP_001098797.1	54.41
*Gallus gallus*	XP_422069.3	54.81
*Mus musculus*	NP_034648.2	55.15
*Numida meleagris*	XP_021254294.1	55.19
*Coturnix japonica*	XP_015724280.1	55.19
*Nanorana parkeri*	XP_018408077.1	58.27
*Rana temporaria*	XP_040214133.1	58.74
*Danio rerio* IGFBP5b	NP_001119935.1	78.07
*Danio rerio* IGFBP5a	NP_001092224.1	69.37
*Lates calcarifer* IGFBP5b	XP_018552191.1	94.91
*Lates calcarifer* IGFBP5a	XP_018516555.1	64.68
*Seriola dumerili* IGFBP5b	XP_022596386.1	97.38
*Seriola dumerili* IGFBP5a	XP_022617333.1	65.06
*Larimichthys crocea* IGFBP5b	XP_010732591.1	92.54
*Larimichthys crocea* IGFBP5a	XP_010740884.1	62.59
*Tachysurus fulvidraco* IGFBP5b	XP_027020371.1	70.22
*Tachysurus fulvidraco* IGFBP5a	XP_026989260.1	60.15
*Ctenopharyngodon idella* IGFBP5b	APX54482.1	77.53
*Ctenopharyngodon idella* IGFBP5a	APX54481.1	71.59

### The expression profiles of TroIGFBP5b were regulated by *V. harveyi* infection

**Figure 2:**
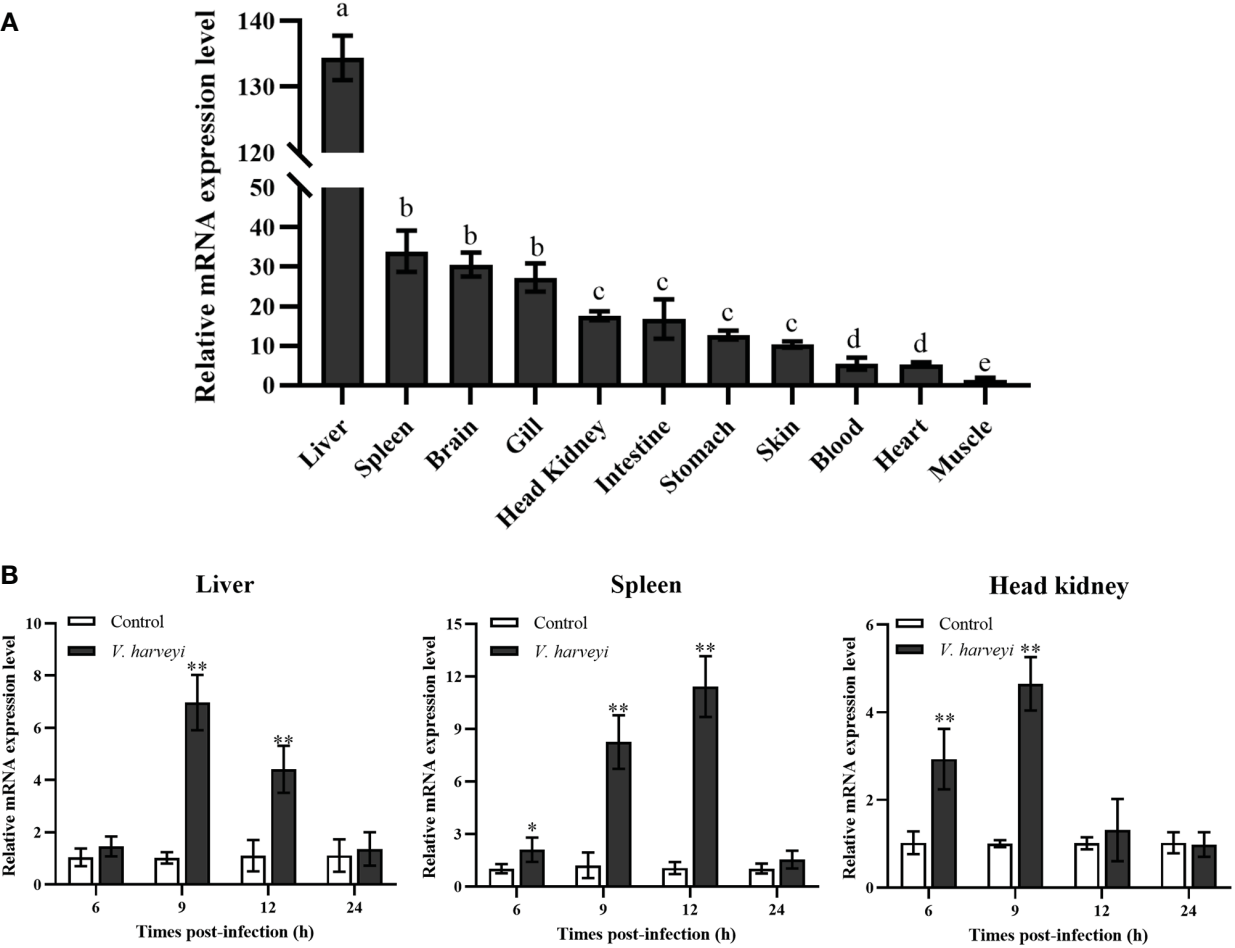
Relative mRNA expression levels of *TroIGFBP5b*. **(A)** The mRNA expressions of *TroIGFBP5b* were detected in 11 tissues using qRT-PCR, and the muscle was set as 1. Groups with the same letters are not significantly different from each other (*P* < 0.05). **(B)** The IGFBP5 expressions of different times infected with *V. harveyi* in the liver, spleen, and head kidney were determined by qRT-PCR, and phosphate-buffered-saline-injected group—as the control—was set as 1. *Beta-2-microglobulin* was used as a reference gene in normalizing. Data were presented as means ± SD (*N*, number of fish used; *N* = 3). *P < 0.05, **P < 0.01.

The expression pattern of *TroIGFBP5b* in *T. ovatus* was detected in 11 tissues using qRT-PCR: heart, stomach, brain, liver, skin, head kidney, intestine, spleen, gills, blood, and muscle. The results showed that it was the lowest in the muscle, set as 1. In contrast to the set criterion of muscle, the expression levels of the other tissues ranking from high to low were as follows: liver (134.44-fold), spleen (33.19-fold), brain (30.43-fold), gill (27.51-fold), head kidney (17.69-fold), intestine (17.18-fold), stomach (12.67-fold), skin (10.12-fold), blood (5.51-fold), and heart (5.11-fold) (**Figure 2A**). The results for the expression of *TroIGFBP5b* during *V. harveyi* infection in the three main immune organs all displayed a significant enhancement with a similar expression pattern, which tended to decrease after an initial increase. The time points at which the peaks appeared were different. In the liver, the expression peak was at 9 hpi with a 6.86-fold change, and it was also at 9 hpi with a 4.64-fold change in the head kidney. For the spleen, the highest expression (10.93-fold) was observed at 12 hpi (**Figure 2B**).

### *In vivo* antibacterial ability after TroIGFBP5b overexpression and knockdown

**Figure 3:**
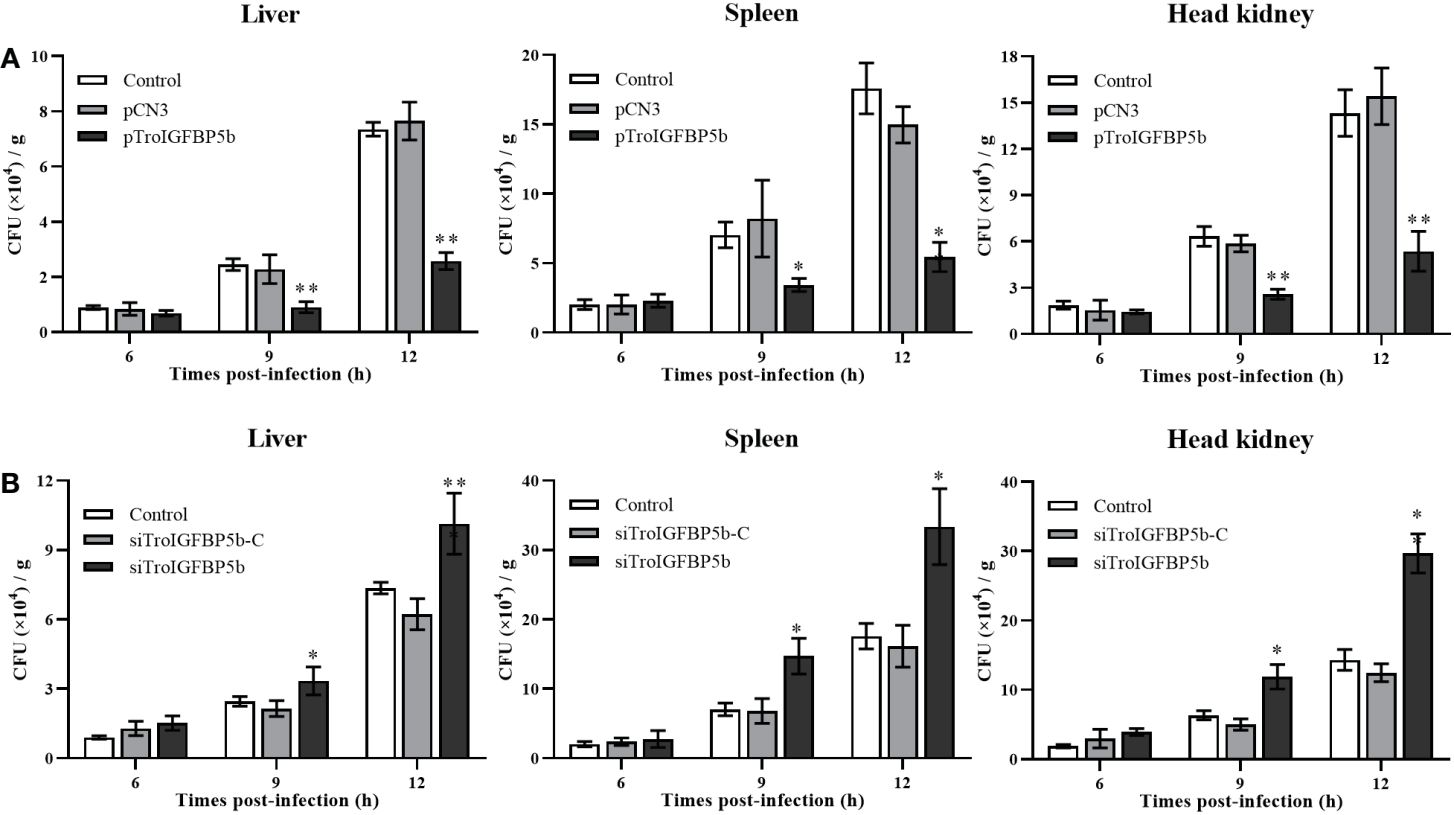
Antibacterial ability after TroIGFBP5b overexpression and knockdown. The bacteria colony counts in the liver, spleen, and head kidney were detected after the overexpression plasmid was injected for 5 days **(A)** and siRNA was injected for 12 h **(B)**. Data were shown as means ± SD (*N* = 3), and the statistical significance was indicated. *P < 0.05, **P < 0.0*1*.

To explore the function of TroIGFBP5b in response to bacterial infection, TroIGFBP5b was overexpressed *in vivo* by injection with pTroIGFBP5b, pCN3, or PBS (as a control). On the 5th day after the plasmid injection, the expression levels of TroIGFBP5b in the liver, spleen, and head kidney of fish treated with pTroIGFBP5b were significantly higher than in the control using qRT-PCR analysis, indicating that the overexpression of TroIGFBP5b was successful (**Supplementary Figure S2**). In the liver of the pTroIGFBP5b overexpression group, the bacterial load decreased by 1.7- and 1.85-fold compared with that of the control at 9 and 12 hpi, respectively. In the spleen, it was decreased by 1.05-fold and 2.23-fold in the pTroIGFBP5b group compared with the control group at the two time points. Furthermore, the pTroIGFBP5b group had an approximately 1.46- and 1.67-fold reduction in head kidney bacterial load at 9 and 12 hpi, respectively (**Figure 3A**).

*In vivo* siRNA technology was used to further analyze the effect of TroIGFBP5b against pathogen infection. The qRT-PCR analysis showed that the expression levels of TroIGFBP5b in the liver, spleen, and head kidney of fish treated with pTroIGFBP5b were significantly decreased than in the control group using qRT-PCR analysis, indicating that the knockdown of TroIGFBP5b was successful after siRNA injection for 12 h (**Supplementary Figure S3**). After being challenged by *V. harveyi*, the liver bacterial counts were approximately 1.90-, 1.50-, and 1.55-fold higher in the siTroIGFBP5b-injected group at 6, 9, and 12 hpi than in the control group, respectively. On the other hand, at 6, 9, and 12 hpi, the splenic bacterial load after siTroIGFBP5b injection was approximately 1.97-, 1.87-, and 1.69-fold higher than that in the control group. In the head kidney, the bacterial loads increased by 1.63-, 1.54-, and 1.94-fold compared with the control at 6, 9, and 12 hpi, respectively (**Figure 3B**).

### Subcellular localization of TroIGFBP5b WT and HBM- and SP-deficient mutants

**Figure 4:**
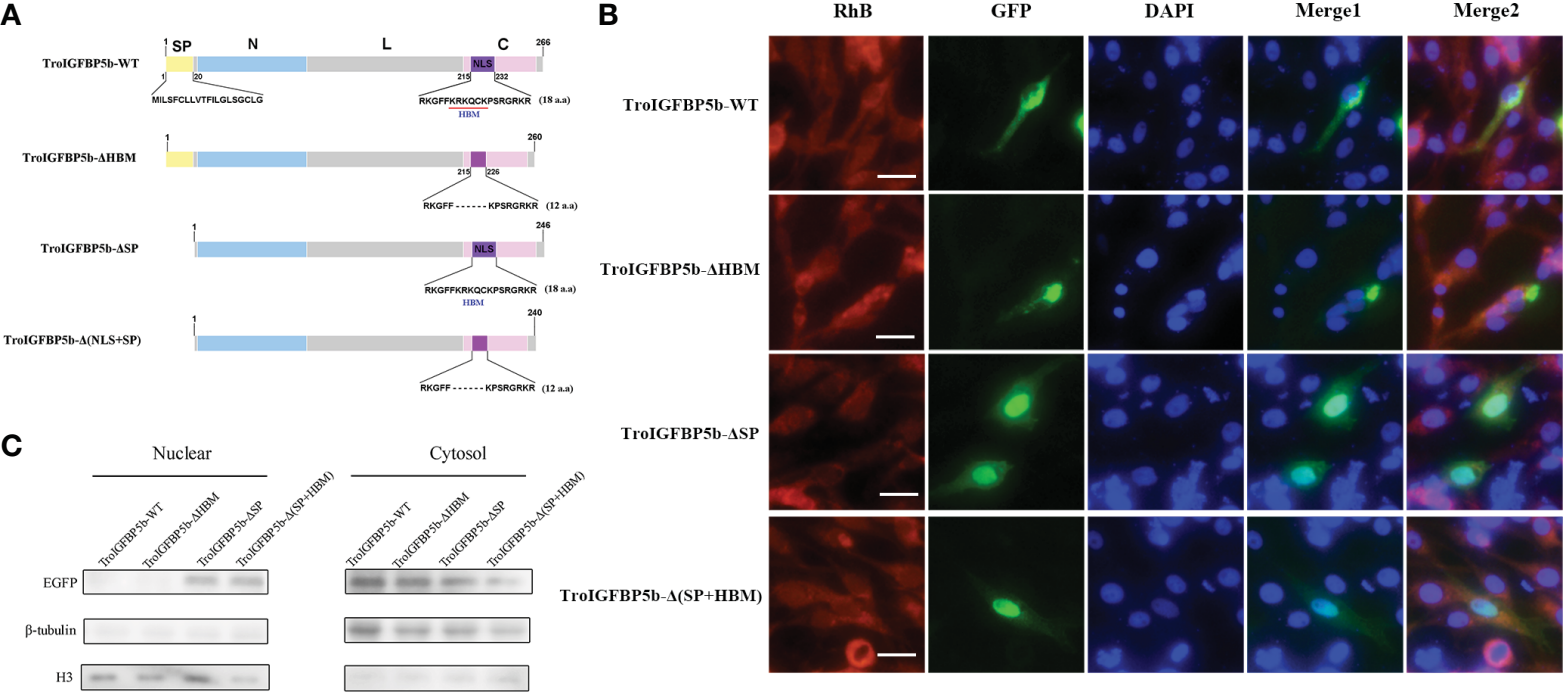
Subcellular localization of TroIGFBP5b and its mutants in golden pompano snout (GPS) cells. **(A)** Schematic diagrams of the structural domain comparison between the TroIGFBP5b wild type (TroIGFBP5b-WT), heparin-binding motif (HBM)-deleted TroIGFBP5b (IGFBP5-ΔHBM), signal peptide (SP)-deleted TroIGFBP5b (IGFBP5-ΔSP), and both HBM- and SP-deleted TroIGFBP5b [IGFBP5-Δ(HBM+SP)]. Different colors represented different structural domains. **(B)** GPS cells transfected with pTroIGFBP5b-N3 and mutants were observed under fluorescence microscopy. Bar = 20 μm. **(C)** Western blot analysis of the nuclear and cytoplasmic protein extracted from the cells mentioned above.

As mentioned above, TroIGFBP5b contains an SP sequence and an HBM in its NLS domain. To explore the role of these two motifs in the subcellular localization characteristics of TroIGFBP5b, recombinant plasmids containing TroIGFBP5b wild type, HBM-deleted TroIGFBP5b, SP-deleted TroIGFBP5b, and both HBM- and SP-deleted TroIGFBP5b were constructed based on pEGFPX-N3, which were named TroIGFBP5b-WT, TroIGFBP5b-ΔHBM, TroIGFBP5b-ΔSP, and TroIGFBP5b-Δ(HBM+SP), respectively (**Figure 4A**). GPS cells were transfected using LipoFiter3.0. The inverted fluorescence microscopy results showed IGFBP5-WT and IGFBP5-ΔHBM to be mainly observed in the cytoplasm, indicating that TroIGFBP5b might be localized in the cytoplasm of GPS cells. While in the absence of the SP sequence its localization changed, the main localization was transferred from the cytoplasm to the nucleus (**Figure 4B**). The protein level displayed by the green fluorescence was consistent with those observed by fluorescence microscopy (**Figure 4C**).

### IGFBP5-ΔHBM altered the intracellular actions of TroIGFBP5b *in vitro*

**Figure 5:**
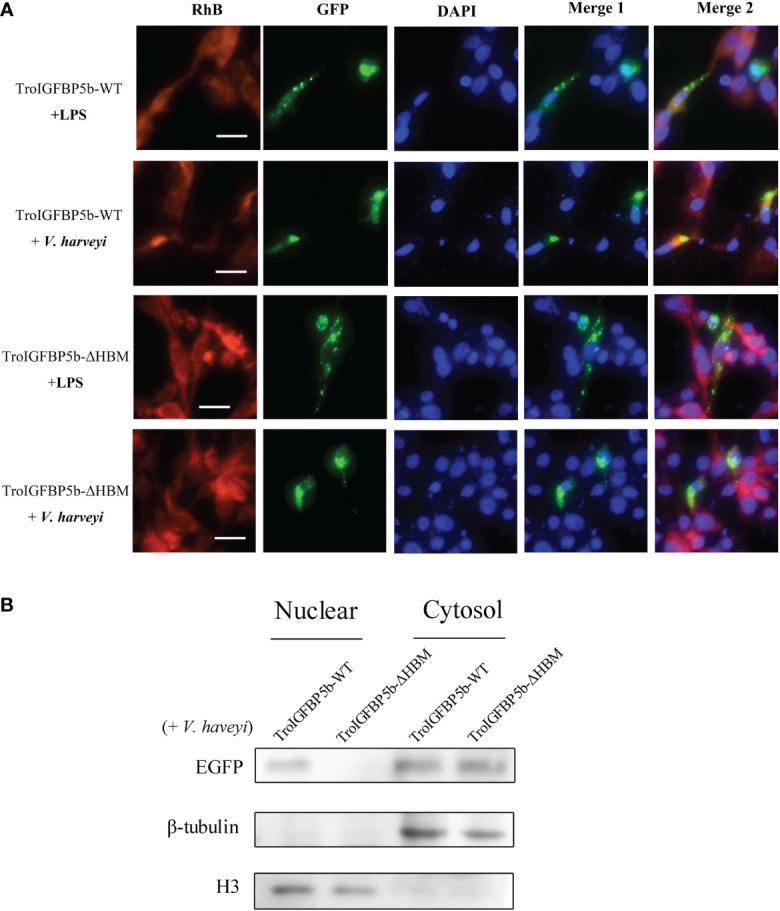
IGFBP5-ΔHBM subcellular localization after stimulations. **(A)** After being transfected with pTroIGFBP5b-WT-N3 and pTroIGFBP5b-ΔHBM-N3 for 48 h, golden pompano snout cells were stimulated with lipopolysaccharide and *V. haveyi*. Bar = 20 μm. **(B)** The Western blot analysis of the nuclear and cytoplasmic protein extracted from the cells mentioned above was stimulated by *V. haveyi* for 4 h.

Although the subcellular localization of IGFBP5-WT and IGFBP5-ΔHBM did not seem different through observation, we wondered if the HBM deficiency would affect the subcellular localization under pathogen stimulation. To explore this postulate, GPS cells transfected with TroIGFBP5b-WT and TroIGFBP5b-ΔHBM were treated with LPS or *V. harveyi*. According to the results, green fluorescence could be observed in the nucleus after stimulation in the TroIGFBP5b-WT transfected cells. However, the cells transfected with TroIGFBP5b-ΔHBM did not undergo such transfer, suggesting that the deletion of HBM would result in the loss of nuclear transfer reaction in response to stimulation (**Figure 5A**). We also evaluated the green fluorescence at the protein level, and the results were in agreement with the results mentioned above (**Figure 5B**).

### *In vitro* effects on the lymphocytes and macrophages of rTroIGFBP5b and rTroIGFBP5b-ΔHBM

**Figure 6:**
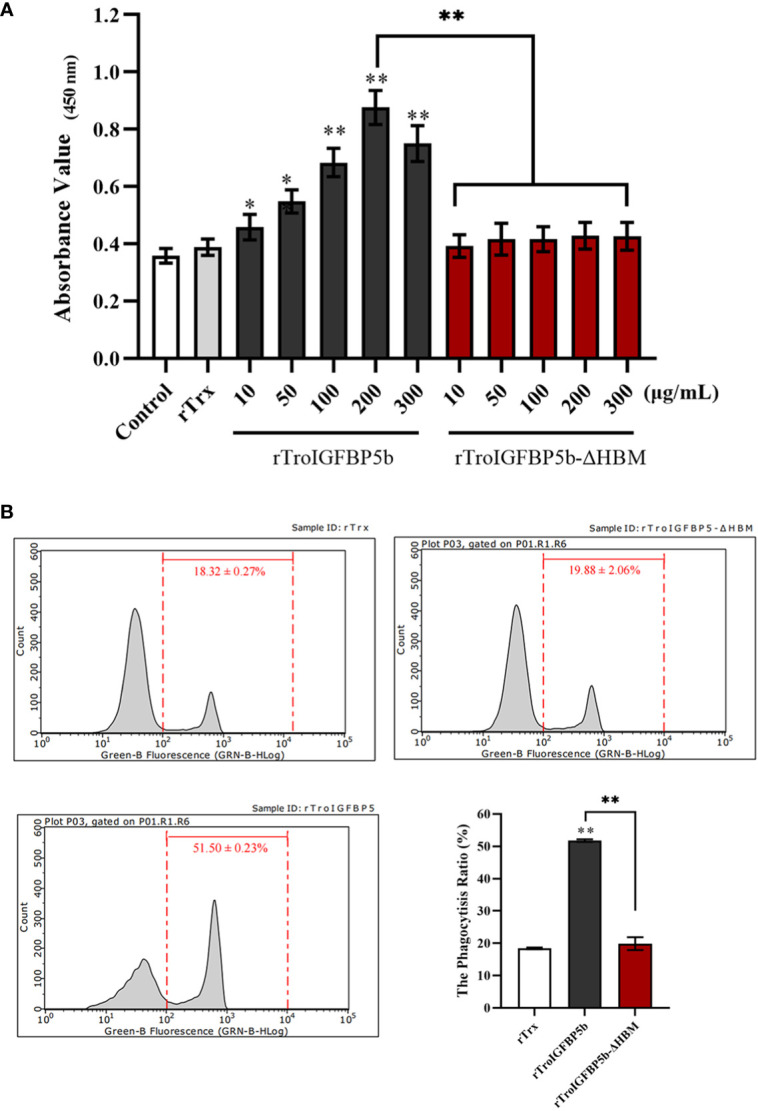
Effect of TroIGFBP5b and heparin-binding motif mutant recombinant proteins on the proliferation of head kidney lymphocytes (HKLs) and phagocytosis of head kidney macrophages (HKMs). **(A)** The proliferation of HKLs was examined by CCK8 assay after incubation with different concentrations of rTroIGFBP5b or rTroIGFBP5b-ΔHBM. **(B)** The phagocytic activity of HKMs treated with recombinant proteins was examined with a flow cytometer after incubating with fluorescent microsphere for 2 h Values are shown as means ± SD (*N* = 3). *N*, number of times the experiment was performed. The statistical significance was indicated (*P < 0.05, **P < 0.01).

Since TroIGFBP5b was involved in defense against the pathogen *in vivo*, we wonder whether TroIGFBP5b had any effect on immune cell activities and if HBM contributed to the processes. To prove this opinion, rTroIGFBP5b, rTroIGFBP5b-ΔHBM, and rTrx (control) were purified, and the lymphocytes and macrophage cells were extracted from the head kidney. According to the CCK-8 assay results, rTroIGFBP5b enhanced HKL proliferation in a dose-dependent manner, and it was shown that 200 mg/ml exhibited the best effect, compared with the cells incubated with PBS and rTrx, while rTroIGFBP5b-ΔHBM had no effects on HKL proliferation (**Figure 6A**). From the results derived by checking using a flow cytometer, it was shown that the phagocytosis rate of the rTroIGFBP5b group was extremely higher than that in the rTrx and rTroIGFBP5b-ΔHBM cells, while the phagocytic activity of those cells that were treated with rTroIGFBP5b-ΔHBM was comparable with that of the rTrx-treated group (**Figure 6B**).

### TroIGFBP5b overexpression could activate the NF-κB pathway, while TroIGFBP5b-ΔHBM could not

**Figure 7:**
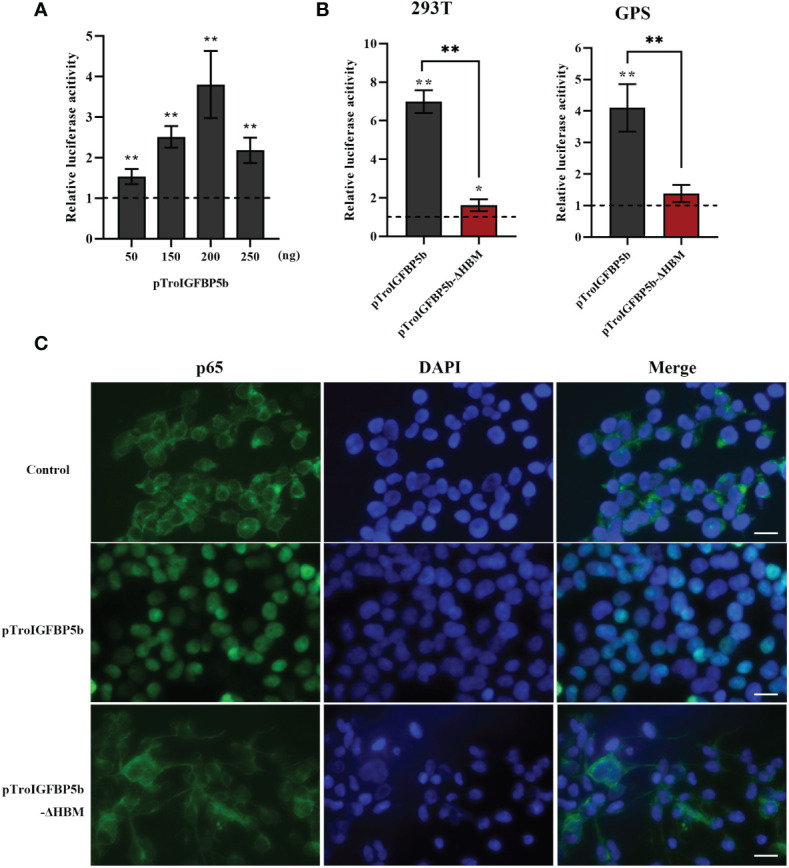
Effects of TroIGFBP5b overexpression on the activity of NF-κB pathways *in vitro*. **(A)** pTroIGFBP5b (50, 150, 200, and 250 ng/well) was co-transfected with pGL4.32 (Luc2P/NF-κB) and pRL-CMV into 293T cells incubated in a 24-well cell plate. **(B)** pTroIGFBP5b (200 ng/well) and pTroIGFBP5b-ΔHBM (200 ng/well) were co-transfected with 200 ng/well pGL4.32 (Luc2P/NF-kB) and 50 ng/well pRL-CMV into 293T and golden pompano snout cells incubated in a 24-well cell plate, respectively. After 48 h post-transfection, firefly and renilla luciferase activities were detected in the cell lysates. The data were shown as means ± SD (*N* = 3). *N*, number of times the experiment was performed. **(C)** 293T cells transfected with pCN3, pTroIGFBP5b, and pTroIGFBP5b-ΔHBM were stained with anti-p65 antibody and AlexaFluor-488. Bar = 30 μm. The experiment was done in triplicate, and one of them was displayed. The statistical significance was indicated (*P < 0.05, **P < 0.01).

A dual-luciferase reporter assay was performed to determine the role of TroIGFBP5b in the NF-κB pathway. It was found that the overexpression of TroIGFBP5b significantly activated the NF-κB luciferase reporter activity in a dose-dependent manner, while the activating effects on the NF-κB signaling did not occur in the TroIGFBP5b-ΔHBM overexpression cells in both 293T and GPS cells (**Figures 7A, B**). This indicated that HBM was important in TroIGFBP5b activation to the NF-κB pathway. The immunofluorescence staining results proved that TroIGFBP5b overexpression promoted the nuclear translocation of p65 in 293T cells. However, this p65 translocation was blocked by overexpressing TroIGFBP5b-ΔHBM in 293T cells (**Figure 7C**). However, due to the poor specificity of the p65 antibody in GPS cells, we did not achieve satisfactory results (data not shown). These results demonstrated that the HBM of TroIGFBP5b seemed to function as a key for activating the NF-κB pathway.

### IGFBP5-ΔHBM lost the antibacterial and activated immune-related gene abilities *in vivo*

**Figure 8:**
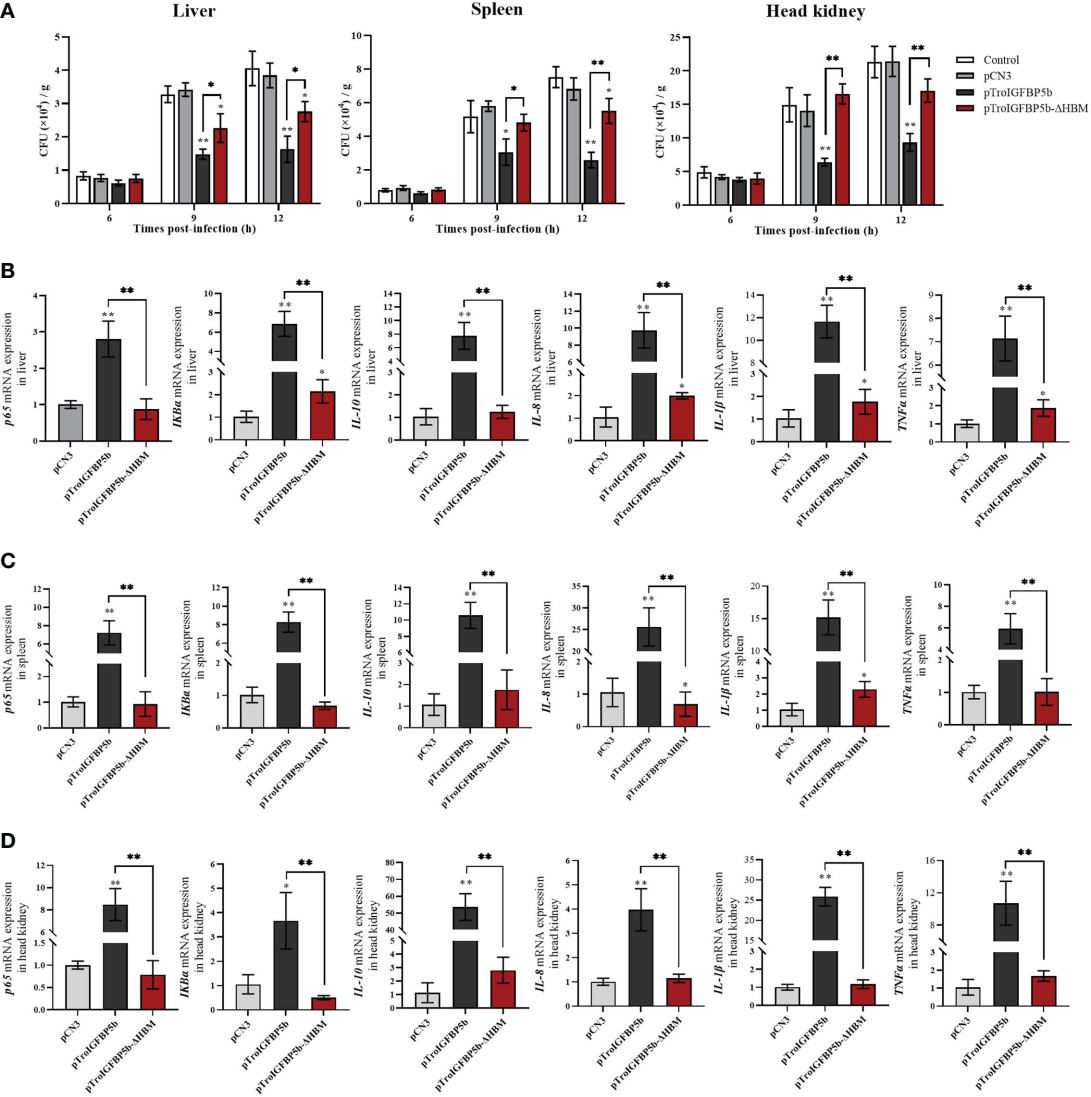
*In vivo* antibacterial ability of IGFBP5-ΔHBM. **(A)** *T. ovatus*, which was injected with pTroIGFBP5b, pTroIGFBP5b-ΔHBM, pCN3, and phosphate-buffered saline (control) for 5 days, was infected with *V. harveyi*; then, the bacterial load in the tissues was determined. Effect of TroIGFBP5b and TroIGFBP5b-ΔHBM on the expressions of proinflammatory cytokines in the liver **(B)**, spleen **(C)**, and head kidney **(D)** at 5 days after the injection of pTroIGFBP5b and pTroIGFBP5b-ΔHBM. The data were shown as means ± SD (*N* = 3), and the statistical significance was indicated (*P < 0.05, **P < 0.01).

Since the HBM sequence could affect the nuclear transfer of TroIGFBP5b protein in response to pathogenic stimulation, we further investigated whether it could affect its antimicrobial activity *in vivo*. Taking the same approach as before, the fish were injected with overexpression plasmids pTroIGFBP5b-WT, pTroIGFBP5b-ΔHBM, pCN3, or PBS (control), respectively. After having been infected with *V. harveyi* for 9 and 12 h, the bacterial loads in the liver, spleen, and head kidney of the pTroIGFBP5b-ΔHBM group were all significantly higher than that in the pTroIGFBP5b group. In the head kidney, the bacterial load in the mutant group was not any different from that of the control group, while in the liver the bacterial load was lower than the control at 9 and 12 hpi, and in the spleen, it was shown to be lower at 12 hpi (**Figure 8A**).

Furthermore, the *in vivo* function mechanism after the overexpression of TroIGFBP5b and TroIGFBP5b-ΔHBM was figured out by examining the expression of immune-related genes using qRT-PCR. According to the results, TroIGFBP5b overexpression could significantly raise the expression level of the selected immune-related genes (p65, *IKBα*, *IL-8*, *IL-10*, *IL-1β*, and *TNF-α*). TroIGFBP5b-ΔHBM could also affect some genes compared with pCN3, such as *TNF-α*, *IKBα*, *IL-8*, and *IL-1β* in the liver and *IL-8* and *IL-1β* in the spleen. However, compared with the TroIGFBP5b group, the upregulated effect on those genes was significantly decreased in TroIGFBP5b-ΔHBM. To sum up, the upregulated expression of immune-related genes induced by TroIGFBP5b was practically shut down in the absence of HBM (**Figures 8B–D**).

## Discussion

IGF is instrumental in growth regulation, development, and immune response (1, 4, 39). Meanwhile, IGFBPs have crucial roles due to their high binding affinity to IGFs (3, 40). In this study, a teleost IGFBP5 of golden pompano, TroIGFBP5b, was cloned, and its expression and biological properties were analyzed accordingly.

Similar to other members of the IGFBP family, TroIGFBP5b was found to have a highly conserved structure containing N- and C-terminal domains, suggesting that IGFBP5 might present as highly conservative during structural and functional evolution (8, 16, 29, 41). Similar to other reported IGFBP5, the NLS sequence of TroIGFBP5b also included a putative classical HBM (^206^KRKQCK^211^), which might be critical in the diverse functions of IGFBP5 (26–28, 42). Due to the teleost-specific whole-genome duplication, some fish were reported to retain two copies of IGFBP5 (IGFBP5a and IGFBP5b), such as zebrafish, grass carp, and Atlantic salmon (8, 16, 17). In this study, the phylogenetic tree results suggested that TroIGFBP5b clustered with IGFBP5b of other teleosts and had the closest phylogenetic relationship with *Lates calcarifer* IGFBP5b. This indicated that IGFBP5 was an evolutionarily conserved protein, and the duplication of the IGFBP5a/b subfamily probably occurred during fish evolution from a genome duplication event. There might be TroIGFBP5ba in the golden pompano genome waiting for us to discover.

A number of studies have reported the tissue-specific expression of IGFBP5. According to these reports, IGFBP5 has been identified in multiple tissues and different types of cells not only in humans and mice (13, 22, 43–46) but also in teleost and invertebrates (8, 16, 19, 47, 48). In mammals, the transcription levels of other IGFBPs were usually more abundant in the liver, while IGFBP5 was different and was more abundant in the kidney (49, 50). In teleosts, the duplicated IGFBP5 showed different expression patterns in the tested tissues. The IGFBP5b mRNA of zebrafish was detected in the brain, gill, eye, heart, gut, kidney, and gonad, while IGFBP5a was detected with a high level in the brain and gill but could not be detected in the liver and muscle (8). Compared with the expression in grass carp, GcIGFBP5b was markedly present in the liver and brain as well as in the heart, skin, and muscles at low levels (16). In the current study, TroIGFBP5b expression was most abundant in immune organs. The top five tissues with the highest expression level were the liver, spleen, brain, gill, and head kidney. In humans, IGFBP5 was closely related to many diseases, such as colorectal cancer, chronic rhinosinusitis, sarcopenia, and so on (51–55), and pathogen infection induced a significantly higher expression of IGFBP5 in mammals—for example, IGFBP5 expression was significantly upregulated after *Salmonella enterica* stimulation in pigs (56). In our study, *V. harveyi* caused a significant induction of TroIGFBP5b in the liver, spleen, and head kidney. All of these suggested that TroIGFBP5b was involved in antimicrobial immunity.

Studies proved that IGFBP5 was a secreted protein, while it was also found in the nucleus to interact with nuclear proteins (23, 44, 57). The nucleus IGFBP5 was detected in the breast cancer cell line (T47D), lung fibroblasts from idiopathic pulmonary fibrosis patients, and vascular smooth muscle cells (25, 41, 58). Various studies also reported that the localization of IGFBP determines its roles, and the NLS domain was crucial for its subcellular location (24, 28, 58, 59). In MDA-MB-435 cells (a kind of breast cancer cell), wild-type IGFBP5 could translocate to the nucleus and inhibit cell proliferation and migration; on the contrary, NLS-mutant was mainly detected in the cytoplasm and enhanced the proliferation and migration of cells (24). In MCF-7 (breast) and LnCaP (prostate) cells, IGFBP2 possessed a functional NLS sequence, and the activation of VEGF expression and subsequent angiogenesis required the nuclear IGFBP2 (60). HBMs are found in many secreted proteins and responsible for binding to heparan sulfate proteoglycans which take part in a variety of biological processes, including signal transduction, cell adhesion, and blood coagulation (61, 62). Furthermore, the HBM in the NLS domain of IGFBP5 C-terminal domain was functional heparin binding motif (42). In teleosts, studies on the relationship between localization and functions of IGFBP are very limited. In zebrafish, the subcellular localizations of IGFBP5a and IGFBP5b were different. IGFBP5a was only found in the nucleus, and IGFBP5b was found in both the nucleus and the cytoplasm (8). Our results showed that TroIGFBP5b with SP was mostly in the cytoplasm, and mature TroIGFBP5b without SP was found in both the nucleus and the cytoplasm. In mammary epithelium, the overexpression of mature IGFBP5 resulted in nuclear localization, whereas upon expression of the secreted form, no nuclear localization was observed under physiological conditions (63). Previous studies proved that the location of protein always affected its role. In the present study, TroIGFBP5b was transferred from the cytoplasm to the nucleus after being stimulated by LPS or *V. harveyi*, while the phenomenon of TroIGFBP5b-ΔHBM that was to be found in the cytoplasm did not occur. Similar findings were found in T47D breast cancer cells, which provided the necessary NLS residues for nuclear accumulation (23). It was interesting that, in mammary epithelium cells, it was found that intracellular trafficking of IGFBP5 would be restricted to vesicular structures in the cytoplasm and not be uptaken into the nucleus unless the integrity of the plasma membrane was compromised (63). In our results, TroIGFBP5b nuclear uptake occurred probably due to the cell membrane damage after LPS or bacteria stimulation, leading to the loss of membrane integrity (64). Our results indicated that HBM plays a key role in the trigger of the TroIGFBP5b translocation to the nucleus upon stimulation.

In mammals, many studies revolved around the function of IGFBP5 in modulating cell migration and proliferation and in regulating immune processes (65). The recombinant IGFBP5 could increase human RPE cell proliferation, promote periodontal tissue regeneration, and reduce local inflammation (43, 66, 67). Multiple studies showed that IGFBP5 could directly affect inflammation mediated by immune cells and suggested that IGFBP5 exerts anti-inflammatory effects by maintaining immune homeostasis (14, 43). In the current study, the biological properties of TroIGFBP5b were analyzed. The *in vitro* assays showed that rTroIGFBP5b enhanced the cell proliferation of PBLs in a dose-dependent manner and significantly enhanced HKM phagocytic activity, suggesting that rTroIGFBP5b could induce the activation of some immune cells. In contrast, rTroIGFBP5b-ΔHBM was significantly reduced in the activation of PBLs and HKMs. Furthermore, *in vivo* overexpression and knockdown experiments confirmed the role of TroIGFBP5b in fish disease resistance. However, after the HBM of TroIGFBP5b was deleted, its antibacterial ability after overexpression was inhibited. These results further confirmed the role of TroIGFBP5b in antibacterial immune response, and HBM played an important role in antibacterial immunity. However, the underlying mechanisms by which IGFBP and HBM participate in immune response remained an area that has not been thoroughly studied and thus needed more research. Relative reports suggested that the IGF system regulated the immune function and represented an important switch governing immune responses (68).

The NF-κB signaling pathway was the main regulator of inflammatory responses to pathogens, and p65, which belonged to the five NF-κB monomers, would translocate from the cytoplasm to the nucleus after activation (69, 70). Nowadays, many studies found that the IGFBP family got its job done *via* the NF-κB signaling pathway, such as IGFBP2, which promotes PDAC cell invasion and metastasis through the NF-κB pathway (69). In prostate cancer cells, rIGFBP-3 significantly suppressed the NF-κB activity (71). IGFBP5 was proven to inhibit the phorbol myristate acetate-induced NF-κB activity and IL-6 expression in U-937 cells (72). The novel findings in this study showed that, in both 293T and GPS cells, TroIGFBP5b showed a significant upregulation effect in NF-κB activity, while overexpression of TroIGFBP5b-ΔHBM did not. Similar results were also discovered in stimulating the transfer of p65 to the nucleus. On the other hand, activating the NF-κB pathway can regulate the expression of related genes, especially inflammatory cytokines (73–75). In our study, the *in vivo* analysis showed that, after the injection with pTroIGFBP5b, the mRNA transcriptions of NF-κB-related genes (p65 and ikB*α*) and several cytokines were significantly induced, including *IL-8*, *IL-10*, *IL-1β*, and *TNF-α*. However, after the HBM of TroIGFBP5b was deleted, its function of upregulating the expression of inflammatory cytokines in immune tissues was almost lost. Therefore, these results demonstrated that TroIGFBP5b could not only activate the NF-κB activity and p65 nuclear translocation but also increase the proinflammatory cytokine level, and this indicated that the HBM domain of TroIGFBP5b seemed to function a key role in these processes.

To sum up, the TroIGFBP5b was cloned and identified in this study. TroIGFBP5b was expressed higher *in vivo* in some immune-related tissues and showed a significant upregulated response after the bacterial infection. Overexpressing TroIGFBP5b could improve the body’s antibacterial immunity significantly. In contrast, this ability was decreased after its knockdown. An HBM-deficient mutation of TroIGFBP5b was constructed to better understand the mechanism of its antibacterial immunity. The *in vitro* studies demonstrated that TroIGFBP5b could promote PBL proliferation, stimulated macrophage activation, induced the NF-κB promoter activity, and promoted the nuclear translocation of p65, while the HBM mutant, compared with the wild type, failed to function with those abilities. Overall, the results showed that TroIGFBP5b was essential in the antimicrobial immunity of golden pompano and that HBM was also of great importance in the NF-κB pathway activation.

## Data availability statement

The datasets presented in this study can be found in online repositories. The names of the repository/repositories and accession number(s) can be found in the article/**Supplementary Material**.

## Ethics statement

The animal study was reviewed and approved by Animal Care and Use Committee of the Hainan University. Written informed consent was obtained from the owners for the participation of their animals in this study.

## Author contributions

HD and YZ conceived and designed the experiments and wrote the manuscript draft. XD, PZ, and ZC performed the experiments and analyzed the data. YS was responsible for forming the hypothesis, project development, data coordination, and writing, finalizing, and submitting the manuscript. All authors contributed to the article and approved the submitted version.
