# Supplementary material for: Insulin-like growth factor binding protein 5b of Trachinotus ovatus and its heparin-binding motif play a critical role in host antibacterial immune responses via NF-κB pathway
Source: Front Immunol. 2023 Feb 14;14:1126843. doi: 10.3389/fimmu.2023.1126843 (PMC9972581; doi:10.3389/fimmu.2023.1126843)
Supplement: Supplementary file 1 [file DataSheet_1.doc]

Supplementary Material

**Insulin-like growth factor binding protein 5b of *Trachinotus ovatus* (*TroIGFBP5b*) and its** **heparin-binding motif (HBM) play a critical role in host antibacterial immune responses via NF-κB pathway**

**Hehe Du^1,2+^, Yongcan Zhou^1,2+^, Xiangyu Du^2^, Panpan Zhang^2^, Zhenjie Cao^2^, Yun Sun^1,2*^**

^1^ State Key Laboratory of Marine Resource Utilization in South China Sea, Hainan University, PR China

^2^ Hainan Provincial Key Laboratory for Tropical Hydrobiology and Biotechnology, College of Marine Science, Hainan University, PR China

* To whom correspondence should be addressed.

Mailing address:

College of Marine Sciences

Hainan University

58 Renmin Avenue

Haikou 570228

PR ChinaPhone and Fax: 86-898-66256125

Email: [syshui207@126.com](mailto:syshui207@126.com) (Y. Sun)

# Table S1 Primers used in this study

| **Primer name** | **Primer sequence (5’-3’)** | **Amplification target** |
| --- | --- | --- |
| TroIGFBP5b-F1 | GATATCGCCACCATGATTCTGAGTTTTTGCCTT | TroIGFBP5b ORF cloning |
| TroIGFBP5b-R1 | GATATCCTCGTTGTTGCTGCTCTCC |
| TroIGFBP5b-F2 | GATATCGCCACCATGTCATACGTGCCGTGC | TroIGFBP5b ORF without signal peptide cloning |
| TroIGFBP5b-F3 | AAAGGATTCTTCCCATCTCGCCGG | TroIGFBP5b-ΔHBM overlap cloning |
| TroIGFBP-R3 | TTTGAAGAATCCTTTTCTGT |
| M13-F | CGCCAGGGTTTTCCCAGTCACGAC | Detection of pEASY-T1 |
| M13-R | CACACAGGAAACAGCTATGAC |
| P68 | CGACTCACTATAGGGGAATTGT | Detection of pET-TroIGFBP5b and pET-TroIGFBP5b-ΔNLS |
| P70 | AAACCCCTCAAGACCCGT |
| His-R | GTGGTGGTGGTGGTGGTG | Detection of pTroIGFBP5b and pTroIGFBP5b-ΔNLS |
| CN-F | CTTGCGTTTCTGATAGGCACCTA |
| CN-R | TGCGGGCCTCTTCGCTATT |
| N3Test-F | ATGGGCGTGGATAGCGGTTTG | Detection of pIGFBP5-WT-N3, pIGFBP5-ΔNLS-N3, pIGFBP5-ΔSP-N3, and pIGFBP5-Δ(NLS+SP)-N3 |
| N3Test-R | AGGTCAGGGTGGTCACGAGGGT |
| TroIGFBP5b-RT-F | TCTCGTGTAATGGCTCTGTCTTTG | qRT-PCR |
| TroIGFBP5b-RT-R | CACCAGCAGATGCCTCGTTT | qRT-PCR |
| Tro-B2M-RT-F | AAGTCAGTCCACCCAAGGTTCA | qRT-PCR |
| Tro-B2M-RT-R | GGGATTTCCATTCCGTTCTTCATG | qRT-PCR |
| Tro-p65-RT-F | AACCACCTCCACTGCCAACCA | qRT-PCR |
| Tro-p65-RT-R | GGAGACCGTGCTTCCCTGAGAA | qRT-PCR |
| Tro-IKBα-RT-F | CCTCCTGAACCTCGGTGCTGAT | qRT-PCR |
| Tro-IKBα-RT-R | AGTTCCTGTGCCGTCTTCTCGT | qRT-PCR |
| Tro-IL-10-RT-F | GTTCGCCTGCCACGCTATGAA | qRT-PCR |
| Tro-IL-10-RT-R | TCCATGTGAGGCTTCAGGTCCA | qRT-PCR |
| Tro-IL-8-RT-F | AGCCTGGGAATGGAGCAGCAT | qRT-PCR |
| Tro-IL-8-RT-R | TCGCAGTGAGAGTTGGCAGGAA | qRT-PCR |
| Tro-IL-1β-RT-F | GGAGACTGTGGAGGACAAGAGC | qRT-PCR |
| Tro-IL-1β-RT-R | GCGGGCAGACATGAAGGTG | qRT-PCR |
| Tro-TNF-α-RT-F | GGCGTCGTTCAGAGTCTCCT | qRT-PCR |
| Tro-TNF-α-RT-R | TCCTCCTGGGCAGTGGTTT | qRT-PCR |
| siTroIGFBP5b-P1 | GGATCCTAATACGACTCACTATAGCTCCTTCCTAAAGACATT | Synthesizing siTroIGFBP5b |
| siTroIGFBP5b-P2 | AAAATGTCTTTAGGAAGGAGCTATAGTGAGTCGTATTAGGATCC |
| siTroIGFBP5b-P3 | GGATCCTAATACGACTCACTATAAATGTCTTTAGGAAGGAGC |
| siTroIGFBP5b-P4 | AAGCTCCTTCCTAAAGACATTTATAGTGAGTCGTATTAGGATCC |
| siTroIGFBP5b-C-P1 | GGATCCTAATACGACTCACTATAGCTCCTTCCTAAAGACATT | Synthesizing siTroIGFBP5b-C |
| siTroIGFBP5b-C-P2 | AAAATGTCTTTAGGAAGGAGCTATAGTGAGTCGTATTAGGATCC |
| siTroIGFBP5b-C-P3 | GGATCCTAATACGACTCACTATAAATGTCTTTAGGAAGGAGC |
| siTroIGFBP5b-C-P4 | AGCTCCTTCCTAAAGACATTTATAGTGAGTCGTATTAGGATCC |

**Fig. S1. The expression verification of *in vivo* overexpression in *T. ovatus*.** pTroIGFBP5b, pCN3, and PBS were injected into the golden pompano respectively. The total RNA of the three tissues taken after 5 d post-injection was extracted, and digested with DNase. The reversed cDNA was used as the template for PCR with primers TroIGFBP5b-RT-F/His-R and Tro-B2M-RT-F/ Tro-B2M-RT-R, respectively. The results for the first time were shown above: the liver (lanes 1, 2, 3), head kidney (lanes 4, 5, 6), and spleen (lanes 7, 8, 9).


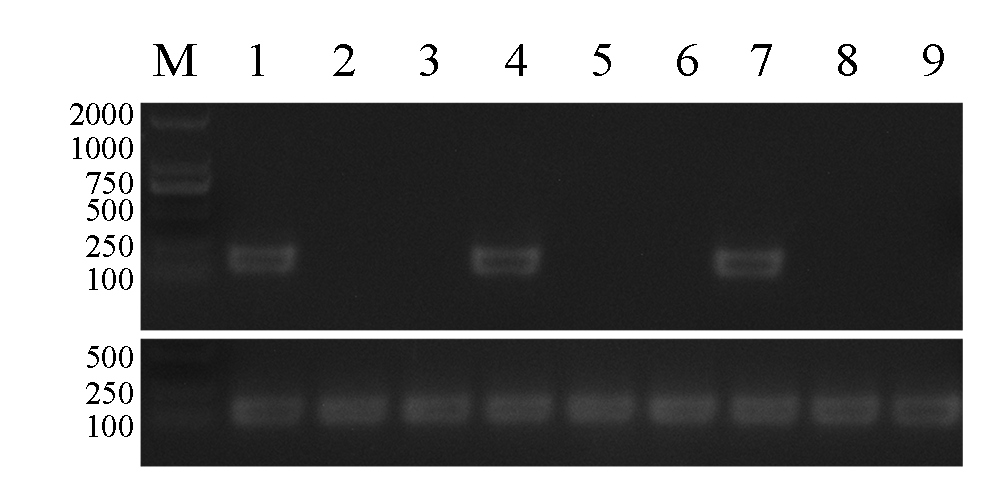


**Fig. S2.** **The expression of plasmid-derived *TroIGFBP5b* in *T. ovatus*.** *T. ovatus* were administered with PBS (as a control), pCN3, and pTroIGFBP5b for 5 days. RNA was extracted from liver, spleen and head kidney for qRT-PCR, the expression level of the control fish was set as 1. Values were shown as means ± SD (N = 3). N, the number of fish used. * *P* < 0.05. ** *P* < 0.01.


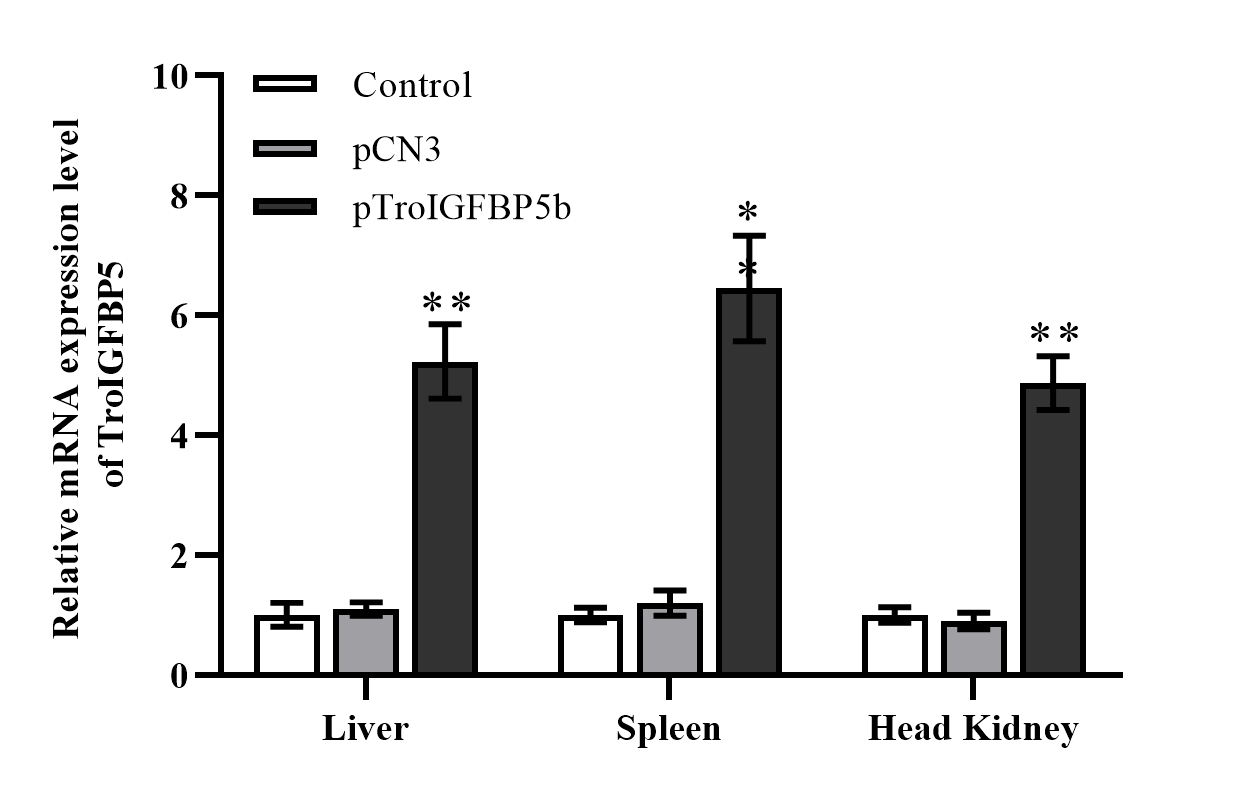


**Fig. S3.** **The expression of knockdown TroIGFBP5b in *T. ovatus*.** *T. ovatus* were administered with PBS (as a control), siTroIGFBP5b-C, or siTroIGFBP5b, and the expression of TroIGFBP5b in liver, spleen and head kidney were determined by qRT-PCR at 12 h post-injection. In those tissues, the expression level of the control fish was set as 1. Values are shown as means ± SD (N = 3). N, the number of fish used. * *P* < 0.05. ** *P* < 0.01.


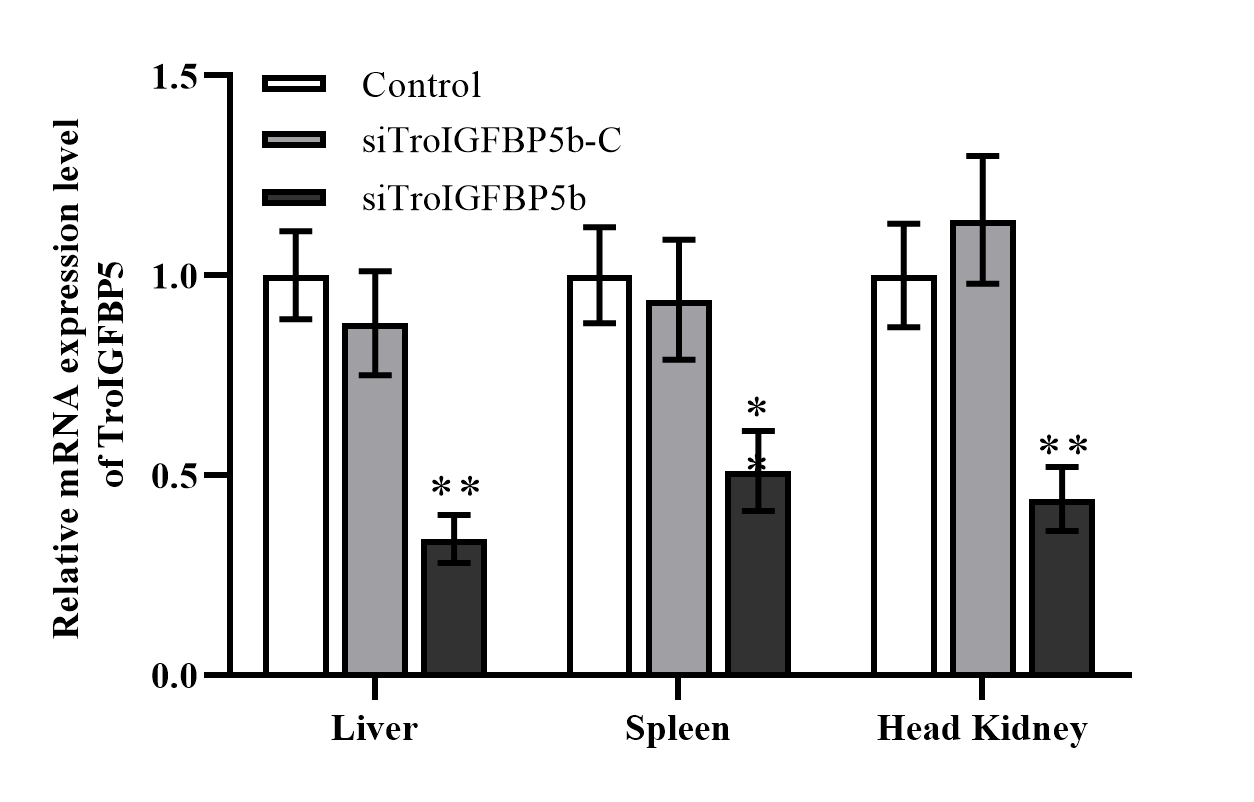


**Fig. S4. SDS-PAGE analyses of purified recombinant proteins of TroIGFBP5b, TroIGFBP5b-ΔHBM mutant, and Trx.** Line M, protein molecular weight marker; lanes 1 and 2, purification of recombinant protein rTrx; lanes and 5, purification of recombinant protein rTroIGFBP5b; lanes 7 and 8, purification of recombinant protein rTroIGFBP5b-ΔHBM.

**
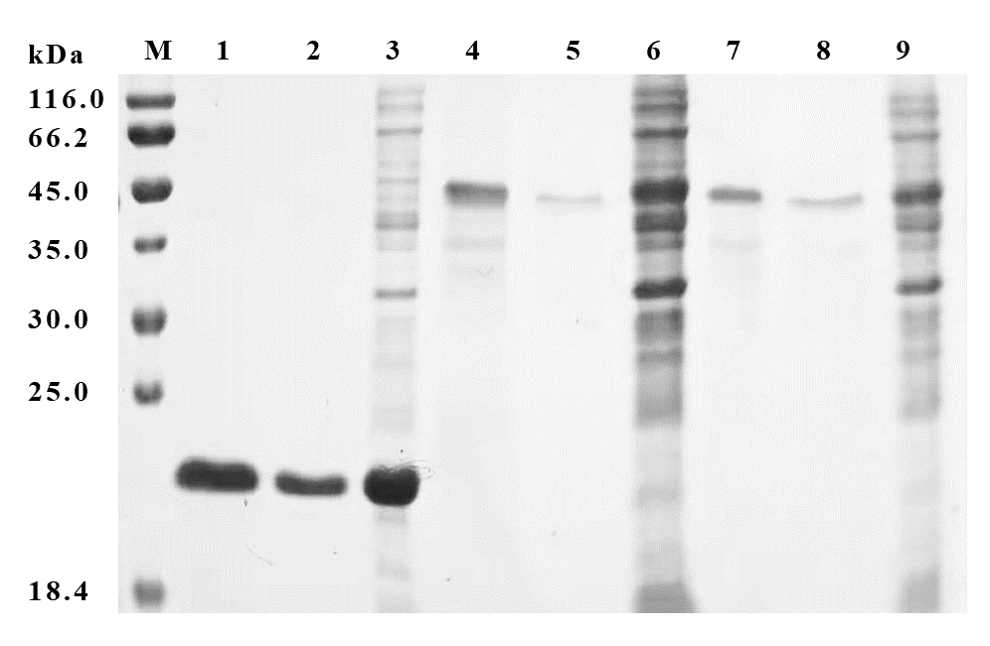
**
